# Oral Administration of Polymyxin B Modulates the Activity of Lipooligosaccharide *E*. *coli* B against Lung Metastases in Murine Tumor Models

**DOI:** 10.1371/journal.pone.0148156

**Published:** 2016-02-01

**Authors:** Jagoda Kicielińska, Agnieszka Szczygieł, Joanna Rossowska, Natalia Anger, Katarzyna Kempińska, Marta Świtalska, Marta Kaszowska, Joanna Wietrzyk, Janusz Boratyński, Elżbieta Pajtasz-Piasecka

**Affiliations:** Institute of Immunology and Experimental Therapy, Polish Academy of Sciences, Wroclaw, Poland; Istituto Superiore di Sanità, ITALY

## Abstract

**Introduction:**

Polymyxin B (PmB) belongs to the group of cyclic peptide antibiotics, which neutralize the activity of LPS by binding to lipid A. The aim of this study was to analyze the effect of PmB on the biological activity of lipooligosaccharide (LOS *E*. *coli* B,rough form of LPS) *in vitro* and in experimental metastasis models.

**Results:**

Cultures of murine macrophage J774A.1 cells and murine bone marrow-derived dendritic cells (BM-DC) stimulated *in vitro* with LOS and supplemented with PmB demonstrated a decrease in inflammatory cytokine production (IL-6, IL-10, TNF-α) and down-regulation of CD40, CD80, CD86 and MHC class II molecule expression. Additionally, PmB suspended in drinking water was given to the C57BL/6 mice seven or five days prior to the intravenous injection of B16 or LLC cells and intraperitoneal application of LOS. This strategy of PmB administration was continued throughout the duration of the experiments (29 or 21 days). In B16 model, statistically significant decrease in the number of metastases in mice treated with PmB and LOS (p<0.01) was found on the 14th day of the experiments, whereas the most intensive changes in surface-antigen expression and *ex vivo* production of IL-6, IL-1β and TNF-α by peritoneal cells were observed 7 days earlier. By contrast, antigen expression and *ex vivo* production of IL-6, IL-10, IFN-γ by splenocytes remained relatively high and stable. Statistically significant decrease in LLC metastases number was observed after the application of LOS (p<0.01) and in the group of mice preconditioned by PmB and subsequently treated with LOS (LOS + PmB, p<0.01).

**Conclusions:**

In conclusion, prolonged *in vivo* application of PmB was not able to neutralize the LOS-induced immune cell activity but its presence in the organism of treated mice was important in modulation of the LOS-mediated response against the development of metastases.

## Introduction

The treatment of patients with Coley's toxin as an enhancer of antitumor immune response is still being re-examined, becoming simultaneously the basis for the analysis of tumoricidal activity of different biological compounds. The mechanism of action of these compounds is considered to be associated with activation of macrophages and vascular endothelial cells, which leads to induction of cellular infiltration of the tumor tissue, cytokine secretion, as well as activation of cell cytotoxic activity against tumors. Nowadays, tumor necrosis factor (TNF) is regarded as the main factor responsible for the therapeutic effect of Coley's toxin preparation [1] which is often supported by interleukins, such as: IL-1, IL-12, IL-15 and IL-18. One of the Coley's toxin components is lipopolysaccharide (LPS) which is believed to possess a strong adjuvant activity in treatment against tumors. For the years of research in murine models a growing body of evidence has shown that the use of LPS can induce antitumor response. Notwithstanding the potential antitumor and/or immunomodulatory activity, LPS can induce numerous side effects and therefore the treatment of tumor bearing patients with LPS has been limited.

A single molecule of LPS consists of three distinct regions: lipid A, the core oligosaccharide and O-specific chain (O-antigen) [2–4]. Lipid A is the region recognized by toll-like receptor 4 (TLR4) expressed on immune cells [5]. Some types of Gram-negative bacteria (eg. *Escherichia coli* B—(*E coli*. B) produce the rough form of LPS (R-LPS), due to the lack of O-antigen region and is referred to as lipooligosaccharide (LOS) [6,7].

One of the antibiotics, which can neutralize the toxic effect of both LPS and LOS is polymyxin B (PmB) [8]. PmB belongs to the group of cyclic peptide antibiotics and shows affinity towards the lipid region of these compounds—lipid A. The amine groups of PmB interact via electrostatic interactions with the phosphate and carboxyl groups of lipid A—Kdo region [8]. PmB is often used in studies involving endotoxin due to two crucial properties of this compound. Firstly, PmB may bind to LPS of many species of bacteria, regardless of their different stereospecificity. This feature allows PmB to be used as a probe for measurement and detection of LPS or lipid A. Secondly, the creation of the PmB-LPS complex can lead to neutralization of the harmful effects of LPS both *in vitro* and *in vivo*. Hence, PmB is used to eliminate the potential LPS contamination in different experimental systems and for the evaluation of activity of potential innate immune cell modulators [9,10]. Adsorbent-based polymyxins are able to purify samples that possess high concentration of different endotoxins, including LPS. Therefore, it is used for hemoperfusion to improve the efficiency of conventional treatment of certain forms of sepsis caused by infection by Gram-negative bacteria [11].

The aim of this study was to analyze the remote influence of polymyxin B on ability of the *E*. *coli* B lipooligosaccharide (LOS) to inhibit lung experimental metastasis. The results suggested that, although prolonged oral application of PmB *in vivo* was not able to elicit strong reactivity of the immune cells, its presence in environment of the LOS-treated mice modulated the trigger of the immune response. Moreover, we postulate, that peritoneal–and/or blood–derived myeloid cells, which responded to LOS administration with the release of the cytokines mobilizing antitumor cells immunity played crucial role in the process. In this context, we suppose that orally administered PmB adjuvant can induce antibacterial intestinal epithelial reactivity, which forces the restriction of prolonged LOS-mediated response.

## Materials and Methods

### Compounds formulations for Lipooligosaccharides (LOS) and Polymyxin B (PmB)

Lipooligosaccharides (LOS) was extracted from *E*.*coli* B according to the procedure described by Galanos [7,12]. The specific method for the extraction of LOS requires mixture of aqueous phenol, chloroform and petroleum ether. Polymyxin B (PmB) (Sigma-Aldrich Chemie GmbH, Germany) was dissolved in drinking water and given to mice in dose of 29.5 mg/l.Both compounds were added in proper concentrations to *in vitro* cultures.

### Cell cultures

#### Melanoma cells (B16) and Lewis Lung Carcinoma cells (LLC)

The B16 mouse melanoma cell line was obtained from ATCC (Rockville, Maryland, USA). The cells were maintained *in vitro* in RPMI-1640 GlutaMAX and Opti MEM GlutaMAX (1:1) (both from Gibco, USA) supplemented with 100 mg/ml streptomycin (Polfa, Poland), 100 U/ml penicillin (Polfa, Poland), 4.5 g/l glucose (Sigma-Aldrich Chemie GmbH, Germany), 0.5% sodium pyruvate (Sigma-Aldrich Chemie GmbH, Germany or HyClone, USA).

The mouse Lewis Lung Carcinoma (LLC) cell line was obtained from ATCC (Rockville, Maryland, USA). The cells were maintained *in vitro* in RPMI-1640 GlutaMAX (Gibco, USA) supplemented with 100 mg/ml streptomycin (Polfa, Poland), 100 U/ml penicillin (Polfa, Poland), 4.5 g/l glucose (Sigma-Aldrich Chemie GmbH, Germany), 0.5% sodium pyruvate (Sigma-Aldrich Chemie GmbH, Germany or HyClone, USA).

Both of cell lines were cultivated in medium supplemented with 10% fetal bovine serum (FBS, Sigma-Aldrich Chemie GmbH, Germany), in standard conditions at 37°C in a humid atmosphere (5% CO_2_).

### Monocyte-macrophage J774A.1 cells and bone marrow-derived dendritic cells (BM-DC) for stimulation with LOS and/or PmB

Cells of the J774A.1 line (ATCC^®^ TIB-67^TM^, Manassas, VA) were cultured in DMEM (ATCC, USA) supplemented with 10% FBS (Sigma-Aldrich Chemie GmbH, Germany) and were passaged 3-times per week. The cells were transferred into 6-well plates (1x10^6^/ml/well) and incubated with the stimulators (LOS or PMB, both at the concentration of 0.1–10 μg/ml) for 24 hours.

Bone marrow–derived dendritic cells (BM-DC) were prepared from bone marrow isolated from female C57BL/6 mice according to the procedure described in previous article [13,14]. The cells were cultured at a density of 1x10^7^ cells/flask in 10ml of RPMI 1640 supplemented with 10% FBS, 40 ng/ml recombinant murine GM-CSF (Invitrogen, Life Technologies Corporation, USA) and 10 ng/ml recombinant murine IL-4 (Immunotools, Germany). After 6 days, BM-DC were transferred into 12-well plates (1x10^6^/ml/well) and maintained for 2 additional days in culture medium with GM-CSF (40 ng/ml) and IL-4 (5 ng/ml). For the last 24h, LOS or PmB (both at the final concentration of 0.1–10 μg/ml) were added to the cultures. After 24 hours, the supernatants from these particular myeloid cultures were collected and stored at 4°C and then they were analyzed using commercially available ELISA sets.

### Phenotypic analysis of BM-DC

BM-DCs were suspended in cold PBS containing 2.5% FBS, washed, and then incubated in a one-step test with the fluorophore-labeled monoclonal antibodies according to the procedure described in previous article [15] (mAbs): hamster anti-mouse APC-CD80 (BD Pharmingen, USA, clone 16-10A1), rat anti-mouse PE-Cy7-CD86 (BD Pharmingen, USA, clone GL1), mouse anti-mouse FITC I-A^b^ (BD Pharmingen, USA, clone 25-9-17), rat anti-mouse RPE-CD40 (BD Pharmingen, USA, clone 3/23), and the appropriate isotype controls: APC-labeled Hamster IgG2,k (BD Pharmingen, USA, clone B83-3), PE-Cy7-labeled Rat IgG2a (BD Pharmingen, USA, clone R35-95), FITC-labeled Mouse IgG2a (BD Pharmingen, USA, clone G155-178), R-phycoerythrin (RPE)-labeled IgG2a (BD Pharmingen, USA, clone R35-95). The cells were stained for 45 min at 4°C. The analysis was carried out using Becton Dickinson FACSCalibur apparatus.

### Ethic statement

Animal care and use were conducted in accordance with the “Interdisciplinary Principles and Guidelines for the Use of Animals in Research, Marketing and Education” issued by the New York Academy of Sciences Ad Hoc Committee on Animal Research and were approved by the 1^st^ Local Ethics Committee for Experiments with the Use of Laboratory Animals, Wroclaw, Poland (authorization number 48/2008). Six to nine-week-old C57BL/6 (B6) female mice were housed under specific pathogen-free (SPF) conditions in a temperature-controlled room with proper darkness-light cycles, fed with a regular diet, and maintained under the care of the Animal Breeding Centre of the Institute of the Immunology and Experimental Therapy. Mice were sacrificed by cervical dislocation.

### Animals and treatment

Mice were obtained from the Center of Experimental Medicine of the Medical University of Bialystok (Bialystok, Poland). In two models of growing B16 or LLC tumors, PmB suspended in drinking water (29.5 mg/l) was given tosix to nine-week-old C57BL/6 (B6) female mice. Administration of the antibiotic began five or seven days respectively prior to the injection of B16 or LLC cells and continued throughout the duration of the experiments. Mice were intraperitoneally (*i*.*p*.) injected with LOS (1 μg/mouse) one hour before the intravenously (*i*.*v*.). inoculation with 2x10^5^ B16 cells or 3x10^5^ LLC cells (collected from the *in vitro* stock culture) in 0.2 ml of Hanks medium/mouse. Experiments were terminated 21 days after inoculation with B16 cells or 15 days after inoculation with LLC cells. During the experiments, mice body weight and temperature were controlled, but no changes were observed. In the B16 model, mice were sacrificed by cervical dislocation on the 7^th^, 14^th^ and 21^st^ day. In the LLC model, mice were sacrificed by cervical dislocation on the 15^th^ day. Experimental metastatic lung colonies from mice were counted under a dissecting stereomicroscope and spleens from 3 mice per group were collected for immunological analyses. The weight of lung obtained from mice were measured. Additionally, in the second experiment with the B16 model peritoneal cells from 4 mice of each experimental group were harvested. Moreover, all mice in experiment were bled to assess and the concentrations of cytokine.

### Stimulation of splenocytes and peritoneal cells

Splenocytes obtained from treated mice (2x10^6^ cells/ml) were stimulated with ConA (0.5 μg/ml) or LOS (1 μg/ml) for 48 hours. Peritoneal macrophages (1x10^6^ cells/ml) were stimulated with LOS (1 μg/ml) for 24 hours. After those time supernatants were collected from above cultured cells.

### Phenotypic analysis of spleen cells

Splenocytes obtained from control or treated tumor bearing mice were liquid-nitrogen frozen and thawed before tests. Then, for lymphoid cell analysis, they were stained in a one-step test with the following fluorophore-labeled anti-mouse monoclonal antibodies (mAbs): anti-CD4-APC (BD Pharmingen, USA, RM4-5), anti-CD8-PE-Cy7 (BD Pharmingen, USA, 53–6.7), anti-CD49b-PE (BD Pharmingen, USA, DX5) and anti-CD19-FITC (BD Pharmingen, USA, 1D3). Phenotype analysis was carried out using the Becton Dickinson FACSCalibur apparatus with Cell Quest Software. For myeloid cell characteristics, the flow cytometry analysis of MDSC surface phenotype was performed as described previously [16] using fluorophore-labeled anti-mouse mAbs: anti-CD11b-PerCP-Cy5.5 (BD Pharmingen, USA, M1/70), anti-B220-APC (BD Pharmingen, USA, RA3-6B2), anti-Ly6G-APC-Cy7 (BD Pharmingen, USA, 1A8), anti-Ly6C-PE (BD Pharmingen, USA, AL-21) and anti-MHCII-FITC (BD Pharmingen, USA, 25-9-17). The cells were stained for 45 min at 4°C. The viability of spleen cells was assessed by incubation with DAPI dye. The analysis was carried out using Becton Dickinson FACSFortessa apparatus with FACSDiva software.

### Phenotypic characteristic of macrophages

Peritoneal macrophages obtained from control and treated mice were stained in a one-step test with the following fluorophore-labeled monoclonal anti-mouse mAbs: anti-F4/80-APC (BD Pharmingen, BM8), anti-CD11b-PerCP-Cy5.5 (BD Pharmingen, M1/70), anti-MHCII-FITC (BD Pharmingen, 25-9-17) and anti-CD86-PE (BD Pharmingen, GL-1). Phenotype analysis was carried out using the Becton Dickinson FACSCalibur apparatus with Cell Quest Software.

### Evaluation of cytokine concentration

IL-6, IL-1β, IFN-γ, TNF-α, IL-4, and IL-10 concentrations in serum and supernatants were measured using commercially available ELISA kits (BD Pharmingen, eBioscience) in accordance with the manufacturer’s instructions.

### Statistical analysis

Statistical analysis of the results (n_i_≥4–16) was carried out using standard nonparametric tests (Kruskal-Wallis test and Mann-Whitney U test (GraphPad Prism 6)). Results were presented as mean ± SD. The values of *p* < 0.05 were considered as statistically significant.

## Results

### Alterations in *in vitro* activation of myeloid cells as the effect of polymyxin B-mediated neutralization of lipooligosaccharide activity

**Fig 1:**
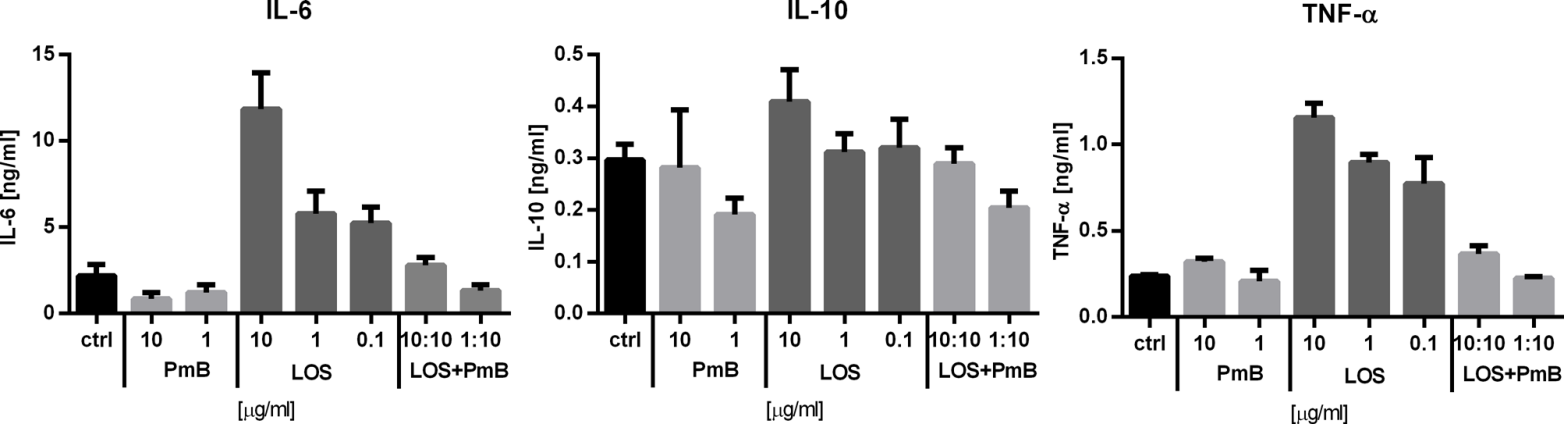
Cytokine production by J774A.1 after LOS and/or PmB stimulation *in vitro*. Cells of J774A.1 line were stimulated with LOS and/or PmB (both at the final concentration of 0.1–10 μg/ml) for 4h (TNF-α) or 24 h (IL-6, IL-10). Combinations of LOS and PmB were used at two ratio: 1:1 (10 μg/ml to 10 μg/ml) or 1:10 (1 μg/ml to 10 μg/ml). Concentration of cytokines in collected supernatants was measured in ELISA assay. To calculate the mean, three sample of each group were analyzed.

In order to evaluate the–antibiotic-mediated neutralization of LPS activity, we estimated the PmB impact on production of IL-6, TNF-α and IL-10 by LOS-stimulated J774A.1 cells. PmB applied alone in concentration of 1 or 10 μg/ml, did not affect the J774A.1 cell activation, whereas while added to the culture simultaneously with LOS it diminished the LOS-related effects in a ratio-dependent manner. A decrease of the cytokine level in culture supernatants was observed both after 4- hour (TNF-α) and 24- hour (IL-6, IL-10) incubation (Fig 1). It should be noted that the neutralizing effect of PmB was visible when its media concentration was at least equal or higher than LOS (ratio of 1:1; this is 10 μg/ml LOS to 10 μg/ml PmB). In such case, a considerable decrease in IL-6 production (2.79 ng/ml) was found compared to 11.83 ng/ml of IL-6 produced by control cells stimulated with 10 μg/ml of LOS. The strongest reduction of the cytokine production (1.34 ng/ml) was found, when the concentration of PmB in the culture was 10-times higher than that of LOS (Fig 1). Likewise in the case of IL-6, the strongest decrease in the TNF-α, as well as IL-10 production was observed at the compound ratio of 1:10 (this is 1 μg/ml LOS to 10 μg/ml PmB). In the 1:10 (LOS to PmB) administration ratio, the production of the cytokines was reduced to 0.29 ng/ml (IL-10) and 0.23 ng/ml (TNF-α), which corresponded to the cytokine concentration levels of the PmB-control group. It should be highlighted that there was no neutralization of LOS activity when the ratio of LOS to PmB in the culture was 5:1 or higher. It was especially visible when the PmB-LOS complexes were prepared outside of the culture and then added to J774A.1 cell cultures (data not shown). Overall, only when PmB concentration in the culture exceeded that of LOS by several times, the antibiotic was able to completely neutralize the LOS activity, which was associated with the decrease in IL-6, TNF-α and IL-10 production.

**Fig 2:**
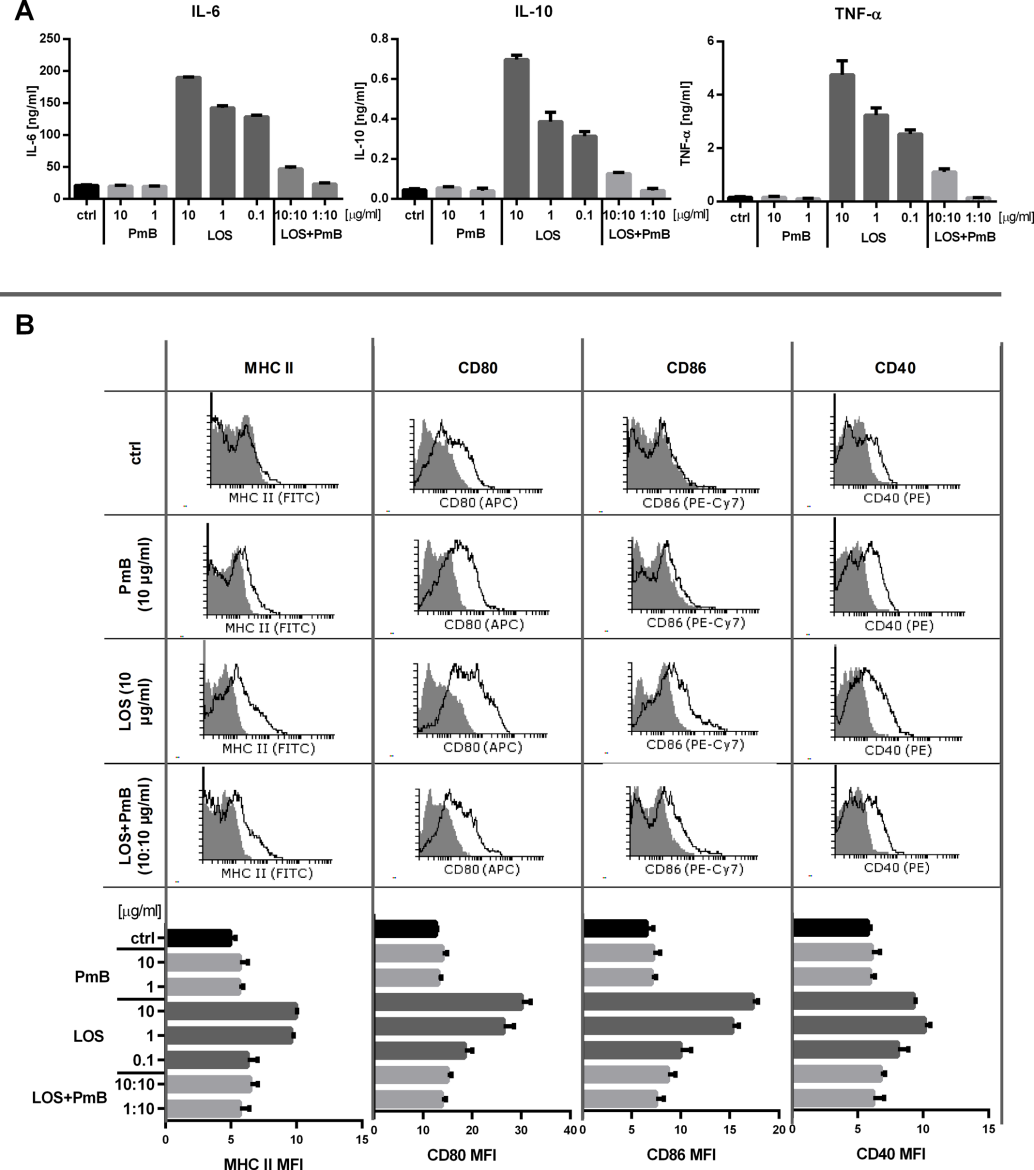
A. Cytokine production by dendritic cells after LOS and/or PmB stimulation *in vitro*. BM-DC obtained from 6-day culture were stimulated with LOS and/or PmB for 24 h (IL-6, IL-10) or 4h (TNF-α). Concentration of cytokines in collected supernatants was measured using ELISA assay. Combinations of LOS and PmB were used in two ratios: 1:1 (10 μg/ml to 10 μg/ml) or 1:10 (1 μg/ml to 10 μg/ml). B. Phenotypic characteristic of dendritic cells after LOS and/or PmB stimulation *in vitro*. Dendritic cells stimulated with LOS and/or PmB for 24h were stained with anti-MHC class II, anti-CD80, anti-CD86 or anti-CD40 antibodies. The figure presents results from one of three independent experiment. The numbers correspond to MFI (mean fluorescence intensity) for the isotype control (gray) vs. the examined surface antigen (black). To calculate the mean, three sample of each group were analyzed.

For evaluation of PmB-mediated inhibition of LOS effects we decided to employ the 7-day bone marrow-derived dendritic cells (BM-DCs) (Fig 2). These cells appeared to be very sensitive to 24 hour stimulation with LOS, which resulted in very high, but still dose-dependent, production of IL-6 (142.2–190.0 ng/ml), TNF-α (3.2–4.7 ng/ml) and even IL-10 (0.39–0.70 ng/ml). Similar to J774A.1 cells, incubation of BM-DCs with PmB did not affect their activity but prevented LOS-mediated cytokine production. The cytokine production was inhibited several or even dozens of times at the administration ratio of 1:1 (10 μg/ml LOS to 10 μg/ml PmB) or completely brought down to the control level when PmB concentration exceeded 10 times the LOS concentration (1 μg/ml LOS to 10 μg/ml PmB) (Fig 2A). Apart from changes in the functional activity, BM-DCs after stimulation with 1 or 10 μg/ml of LOS demonstrated high expression of costimulatory molecules (CD80 and CD86, CD40) as well as MHC class II antigens compared to the control group (Fig 2B). The addition of PmB to LOS-stimulated cells, at the compound ratio of 1:1 prevented the cell surface antigen expression, but it reduced cytokine production to the control level only at compound ratio of 10:1. These data showing the down-regulation of BM-DC-antigen expression and decrease in cytokine secretion in the presence of the excessive PmB concentration and were confirmed our other results. Moreover, PmB-LOS complexes, which were prepared prior to their usage in the ratio of >5:1, appeared to be unable to eliminate LOS activity *in vitro* or activate anti-metastatic response *in vivo* (data not shown).

### PmB-mediated modulation of LOS-inhibition of lung colonization by *i*.*v*. inoculated B16 melanoma-cells

**Fig 3:**
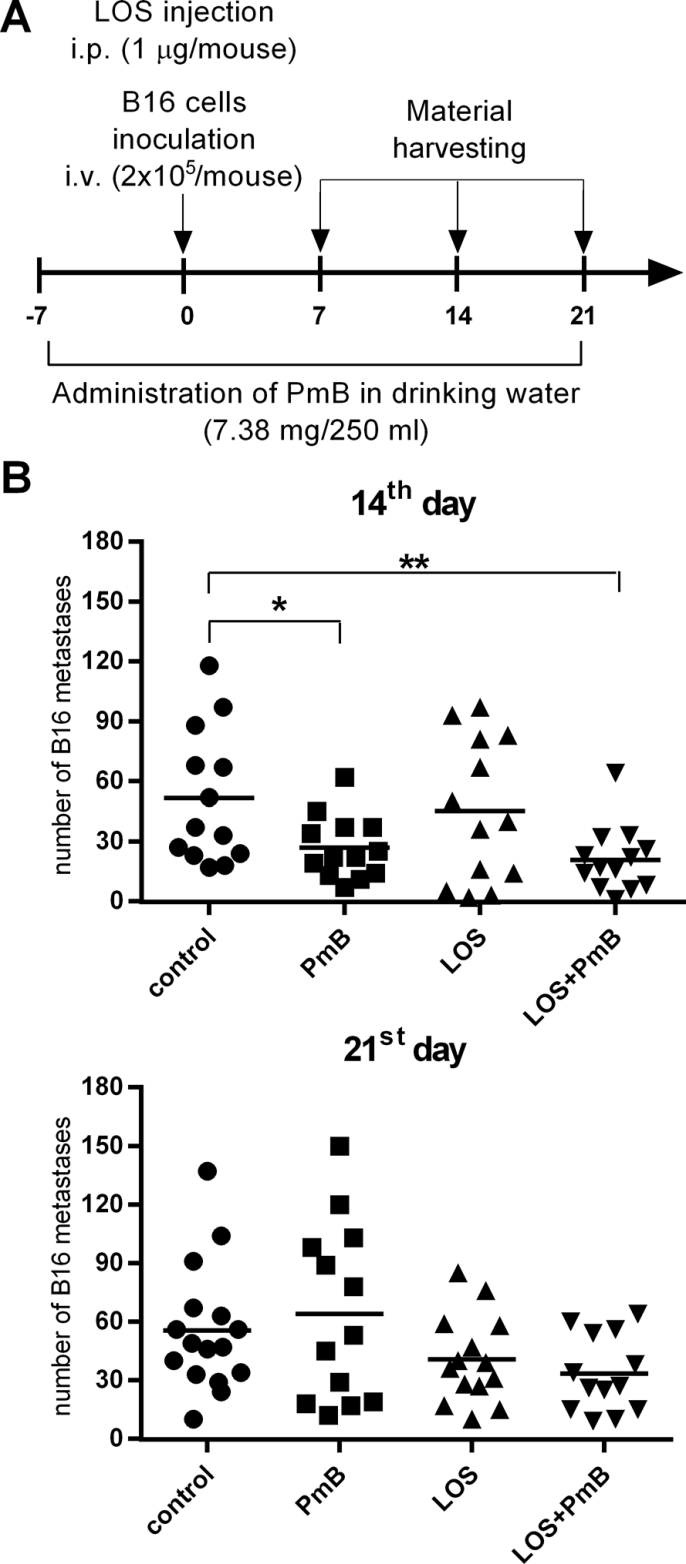
A. Mice treatment schedule. An administration of polymyxin B (7.38 mg/250 ml in drinking water) was started on the 7^th^ day before the i.v. injection of 2x10^5^ B16 cells and continued throughout the experiment duration. Mice were injected with LOS (1μg/mouse) i.p. one hour before cell injection. On the 7^th^, 14^th^ and 21^st^ days after injection, material were harvested and number of lung metastases was determined. On the 7^th^ day metastases were not observed. B. Number of the B16 metastasis in the lung obtained from treated mice on the 14^th^ and 21^st^ day of experiment. To calculate the mean, 13–16 mice of each group of two experiments were tested. The relationship between groups was calculated for n>13 using Mann-Whitney *U* test. differences with a p<0.05 were regarded as significant (*p<0.05, **p<0.01—calculated vs untreated mice).

In respect to our data, based on *in vitro* assays illustrating the PmB-mediated neutralization of LOS effects, as well as the aforementioned study, we decided to use PmB for modulation of the anti-metastatic effect of LOS. For this purpose, PmB was administered in drinking water starting seven days prior to the intravenous inoculation of B16 cells. The main task of the treatment was to reach the highest environmental conditioning before LOS i.p. application (Fig 3A).

The macroscopic metastases were not observed on the 7th day, therefore their number in particular groups was analyzed on the 14th and 21st days of the experiments. The administration of PmB alone, resulted in an approx. 2-fold increase in the metastases’ number on the 21th day in comparison to the upgrowth on the 14th day. Whereas, the application of LOS caused a decrease in the mean number of metastases, especially after a longer period of observation. The most significant decrease in the number of metastases was observed in the group of mice preconditioned by PmB and then treated with LOS (LOS + PmB. On the 14th day of the experiment this effect was statistically significant (p<0.01) compared to the untreated metastases-bearing mice (Fig 3B). However, the further reduction of the metastases’ number that was observed on the 21st day appeared to be statistically insignificant in comparison to the other groups (Fig 3B). The results suggest that exposure of mice to permanent PmB administration had an indirect influence on immune cell response towards LOS.

### Presence of PmB reduces, but not diminishes, the LOS-dependent trigger of peritoneal cell activity

**Fig 4:**
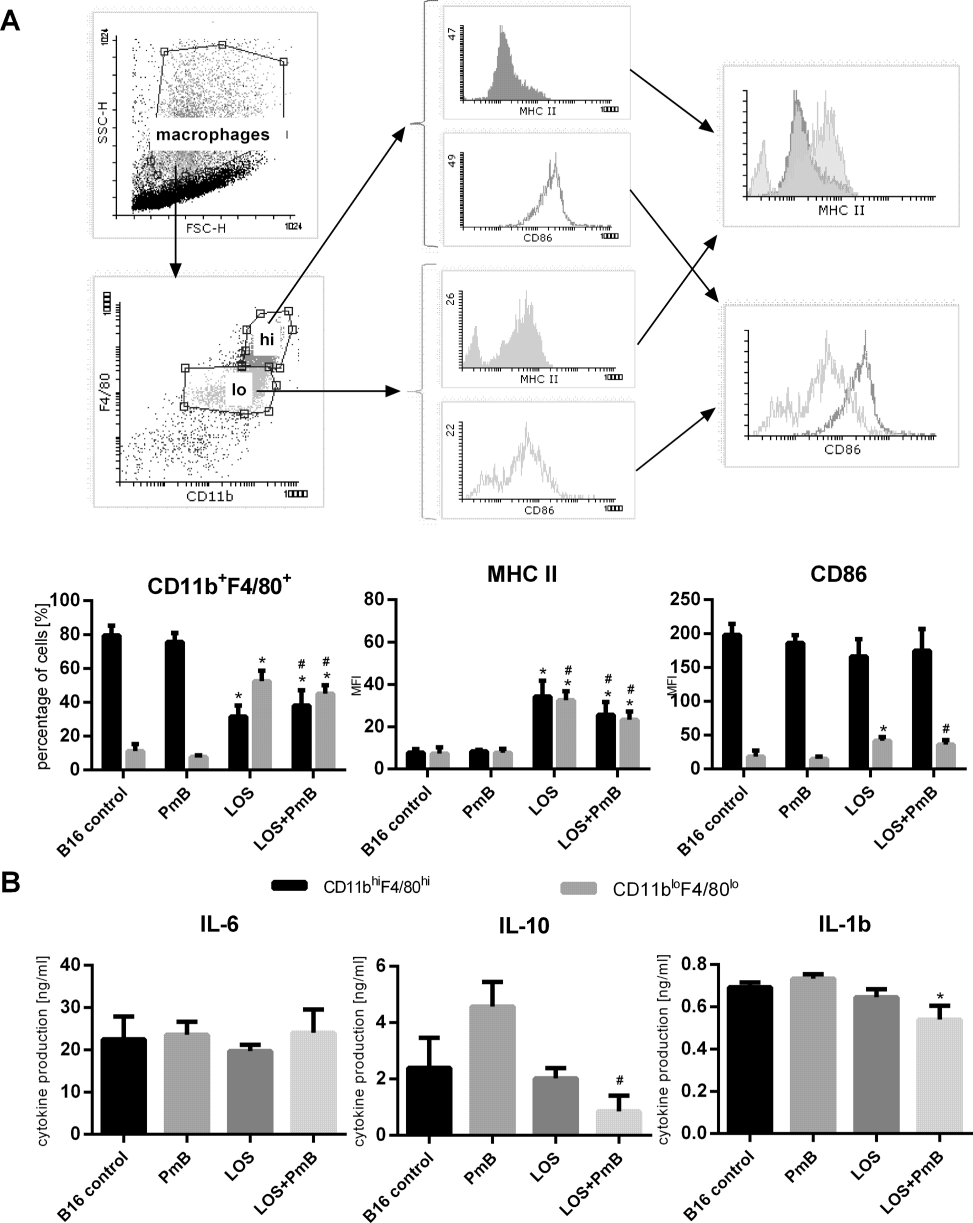
A. Phenotypic characteristics of peritoneal macrophages. Peritoneal macrophages obtained from control or treated tumor bearing mice on 7^th^, 14^th^ and 21^st^ day of experiment were stained in one-step test with following fluorophore-labeled monoclonal anti-mouse antibodies (mAbs): anti-F4/80-APC, anti-CD11b-PerCP-Cy5.5, anti-MHCII-FITC or anti-CD86-PE. Phenotypic characteristics of peritoneal macrophages obtained from healthy mice: CD11b^hi^F4/80^hi^ – 67.23% (CD86 MFI—224, MHC II MFI—9), CD11b^lo^F4/80^lo^ – 11.22% (CD86 MFI—20, MHC II MFI 8) B. Cytokine production by peritoneal macrophages obtained from mice on 7 th day after *in vitro* LOS stimulation. The production of IL-6, IL-10 and TNF-α by stimulated (LOS 1μg/ml, 24h) peritoneal macrophages (1x10^6^ cells/ml) was evaluated by ELISA kits (BD Bioscience). In this supernatants, production of IL-4 was not observed. To calculate the mean four mice of each presented group were tested. Cytokine production by peritoneal macrophages obtained from healthy mice: IL-6–14.45±3.76 ng/ml, IL-10–3.58±1.13 ng/ml, IL-1β – 0.25±0.12 ng/ml. The relationship between groups was calculated for n>13 using Mann-Whitney *U* test. differences with a p<0.05 were regarded as significant (*p<0.05- calculated vs untreated mice, #p<0.05—calculated vs mice treated with PmB).

In order to analyze the relationship between PmB-preconditioned and i.p. LOS administration we harvested the peritoneal cavity cells from tumor-bearing mice on the 7th, 14th, and 21st day (Fig 4A). We divided the peritoneal Mφ into two coexisting subpopulations (Fig 4A) characterized by CD11b^hi^F4/80^hi^ and CD11b^lo^F4/80^lo^ phenotypes respectively. The most significant differences between the controls and groups of mice treated with PmB or treated with LOS +/- PmB were observed on the 7th day of the experiments. In case of the control group and mice exposed to PmB the majority of the peritoneal cells were CD11b^hi^F4/80^hi^ (approx. 80%), whereas less than 20% were CD11b^lo^F4/80^lo^. When mice were treated with LOS, the percentage of CD11b^hi^F4/80^hi^ diminished to 30% (p<0.05—calculated vs untreated mice) and the percentage of CD11b^lo^F4/80^lo^ cells increased to approx. 60% (p<0.05—calculated vs untreated mice). Nevertheless, in presence of both compounds (LOS + PmB) the percentage amounted to 40% of CD11b^hi^F4/80^hi^ (p<0.05—calculated vs untreated mice and treated with PmB) cells and decreased to 50% in case of CD11b^lo^F4/80^lo^ cells (p<0.05—calculated vs untreated mice and treated with PmB). Thus, the effect of LOS stimulation was slightly affected by PmB but not reduced to the level of control or PmB alone. In reference to the information that maturity of cells can be related to down-regulation of expression of F4/80 antigen we further investigated the expression of MHC class II antigens and costimulatory molecule CD86. Seven days after the application of LOS, as well as LOS + PmB, both Mφ subpopulations exhibited high expression of MHC class II antigens and significant increase in expression of CD86 compared to the control and PmB-treated mice (p<0.05). However, these alterations in antigen expressions appeared to be temporary. In case of CD11b and F4/80 antigens, we observed a gradual decrease in percentage of double-positive cells to 30% (after LPS) and 40% (after LOS + PmB treatment), with the expression of MHC-class II on positive cells diminished to approx. 10 MFI (the 21st day).

To determine the peritoneal cell reactivity, the production of IL-6, IL-1β and IL-10 was estimated (Fig 4B). Regardless of the nature of the compounds, peritoneal cells produced cytokines *ex vivo* only after being stimulated with LOS showed the highest differentiation on the 7 day. For all treated groups similar amounts of IL-6 (23–25 ng/ml) and IL-1β (0.6–0.7 ng/ml) were determined. In contrast, treatment with PmB resulted in an increase of IL-10 production to 5 ng/ml, whereas the usage of PmB and LOS diminished IL-10 production to 1 ng/ml (p<0.05 compared to untreated mice). On the subsequent harvesting days the activity of the peritoneal cells decreased. Thus, it is very likely that the newly recruited peritoneal macrophages are responsible for this phenomenon. Any form of treatment of B16 metastases-bearing mice did not change the pro-inflammatory cytokine production rates of peritoneal cells in comparison to control mice. However, usage of PmB elicited the increase in IL-10 production and affected the production mediated by LOS stimulation. Additionaly, no production of IL-4 throughout the experiments was noticed (data not shown).

**Fig 5:**
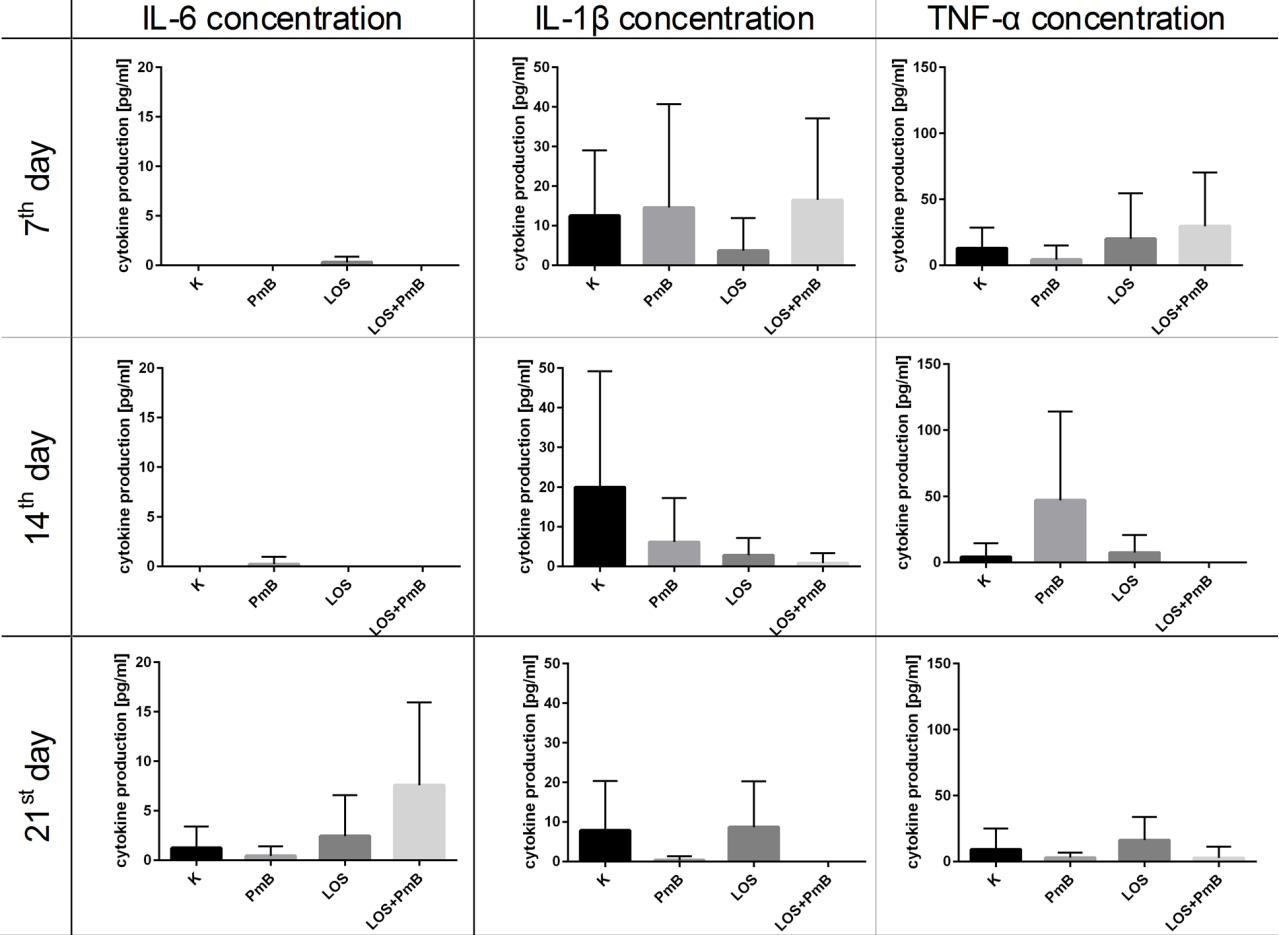
Cytokine concentration in serum obtained from control or treated tumor bearing mice. The concentration of IL-6, IL-1β and TNF-α in murine serum was evaluated by ELISA kits (eBioscience). The presence of the IFN-γ and IL-10 were not observed. To calculate the mean 9–15 mice of each presented group were tested. Cytokine concentration in serum obtained from healthy mice: IL-1β – 5.46 pg/ml; IL-6–0 pg/ml; TNF-α—0 pg/ml.

In order to illustrate the prolonged antimetastatic effect of LOS we decided to check the level of cytokine concentration in sera obtained from treated mice on the 7th, 14th, 21st day (Fig 5). IL-6 was not found on the 7th and 14th day and it appeared in trace amounts on the 21st day, but only in mice which obtained LOS+PmB. In contrast to that cytokine, detectable production of IL-1β or TNF-α was found on the 7th day and in case of mice treated with LOS+PmB their levels diminished on consecutive harvesting days. Our observations of prolonged presence of small concentrations of IL-1β and TNF-α in blood, even 7 days after the usage of LOS (and especially the detection of IL-6 on the 21^st^ day), were most likely the results of a secondary action of myeloid cells in antitumor response than a direct effect of LOS administration. Nevertheless, the presence of the IFN-γ and IL-10 in serum were not noticed (data not shown).

### Effect of PmB on phenotype alterations and induction of the spleen cells from B16 mice model reactivity by LOS administration

**Fig 6:**
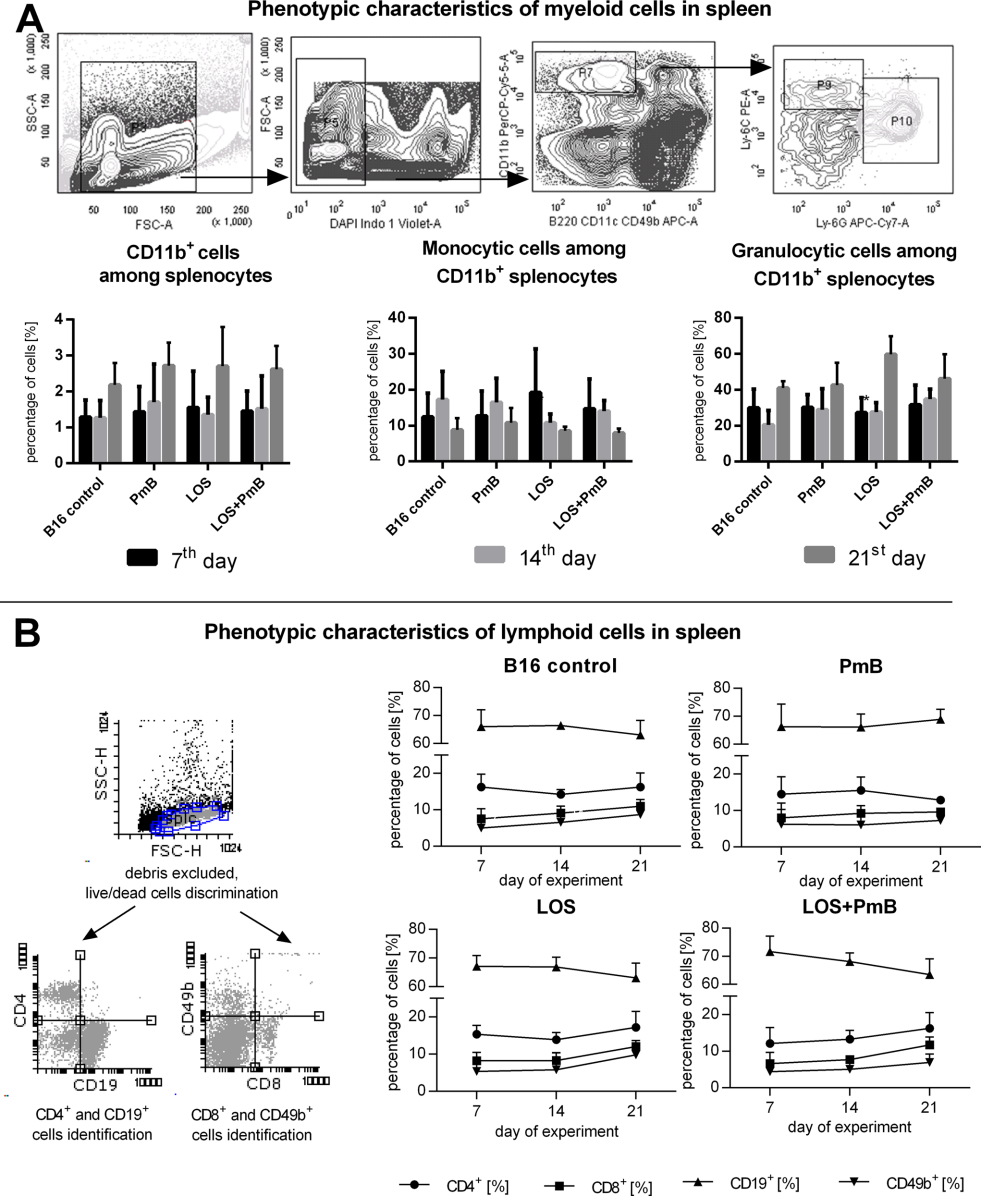
Phenotypic characteristics of splenocytes. A. Phenotypic characteristics of myeloid (A) and lymphoid cells (B) in spleen. Spleen cells from mice treated with LOS and/or PmB were incubated with CD11b-PerCP-Cy5.5, B220-APC, Ly6G-APC-Cy7, Ly6C-PE, MHCII-FITC or anti-CD4-APC, anti- CD8-PE-Cy7, anti-CD49b-PE and anti-CD19-FITC. Phenotype analysis was carried out using the Becton Dickinson FACSCalibur apparatus with Cell Quest Software. To calculate the mean, six mice of each group were tested. The percentages of myeloid cells in healthy mice were: CD11b^+^—0.93%, monocytic cells among CD11b^+^ cells—13%, granulocytic cells among CD11b^+^ cells—28%. The percentages of lymphoid cells in healthy mice were: CD4^+^—13.45%, CD8^+^ 5.95%- CD19^+^—70.6%, CD49b^+^—6.11%.

Regardless of the type of treatment and length of administration time, less than 3% of CD11b^+^ cells were found in spleens (Fig 6A). This population was further divided into Ly6Chi Ly6G^-^ and Ly6C^lo^ Ly6G^hi^ cells. The first subpopulation corresponds to monocyte-derived splenocytes. In mice treated with LOS, it represented approx. 20% of CD11b^+^ cells (the 7^th^ day). On the 14 day of experiment, its size slightly increased, but did not exceed over a dozen percent, in any of the groups. The second subpopulation, which corresponds to granulocyte-derived splenocytes, increased in time-dependent manner and on the 21st day after LOS administration reached 60% of CD11b^+^ cells. Meanwhile, in mice conditioned with PmB and LOS the percentage of Ly6C^lo^ Ly6G^hi^ cells decreased to the level of the PmB-treated group. The question whether CD11b^+^ Ly6C^lo^ Ly6G^hi^ cells would be able to convert into inflammatory effectors or “patrolling” cells or whether the observed changes are associated with recruitment of these cells to an inflamed tissue remains open.

In our experiments no significant changes in the percentage of CD19^+^, CD4^+^, CD8^+^, CD49b^+^ splenic subpopulations (Fig 6B) were revealed. A slight decrease in the percentage of B cells was observed between the 7th and 21st day in mice treated with LOS or LOS + PmB, but not in other groups. Alternatively, in mice that were preconditioned with PmB the percentage of CD4^+^ cells was slightly decreased on the 21st day. After administration of LOS and LOS + PmB, a slight increase in CD8^+^ and CD49b^+^ cell percentage was observed on the 14th and 21st day of experiments in comparison to the control.

**Fig 7:**
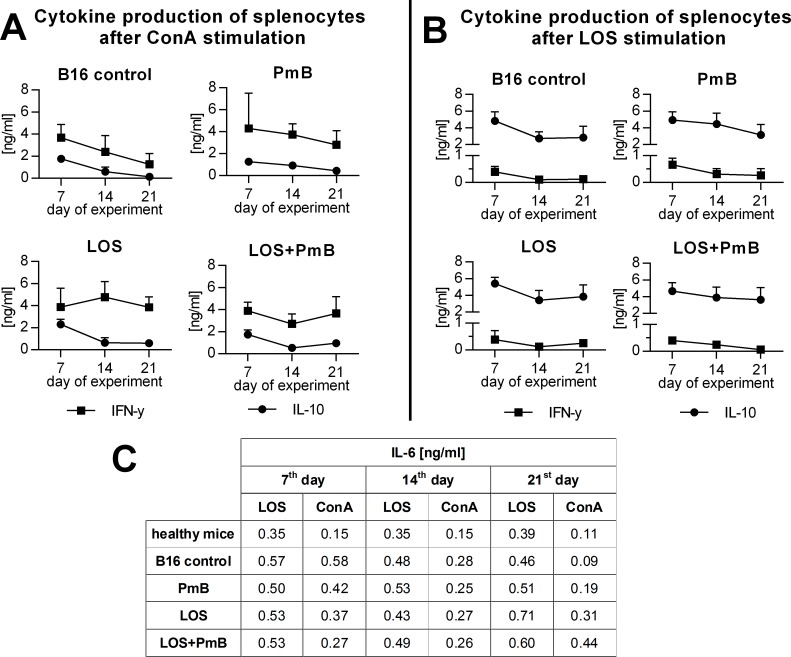
Cytokine production by spleen cells after ConA (A) or LOS (B) stimulation. The concentration of IFN-γ and IL-10 was measured in supernatants collected from above splenocytes (2x10^6^ cells/ml) incubated for 48h with ConA (0.5 μg/ml) or LOS (1 μg/ml), using commercially available ELISA kits (BD Bioscience or eBioscience). To calculate the mean, six mice of each group were analyzed. The production of IL-4 after ConA stimulation was not observed. Cells from healthy mice stimulated with ConA produced 3.53 ng/ml of IFN-γ and 0.89 ng/ml of IL-10. Cells from healthy mice stimulated with LOS produced 0.06 ng/ml of IFN-γ and 3.51 ng/ml IL-10.

Despite minimal alterations in the spleen cell phenotype we have estimated the level of spleen cell reactivity (Fig 7). For this purpose, splenocytes were stimulated *ex vivo* with ConA (Fig 7A and 7C) or LOS (Fig 7B and 7C), and the concentrations of IL-6, IFN-γ, and IL-10 in the supernatants from the aforementioned cultures were measured. The level of IL-6 production proved to be very similar regardless of the type of *in vivo* treatments and types of *ex vivo* stimuli (Fig 7C). Production of IFN-γ was similar in all mice groups. However, on the 14th day, in mice treated with LOS+/-PmB statistically significant (*p*<0.05) changes in cytokine concentration were noticed as compared to the control. In these supernatants the IL-10 concentration amounted to approx. 2 ng/ml on the 7th day and was further reduced regardless of the nature of treatment. This suggests that tumor Ag-specific response of splenic lymphocytes did not occur after a single application of LOS, but on the other hand it triggered the down-regulation of the number of metastases. When splenocytes were stimulated *ex vivo* with the same compound, that was previously administered *in vivo*, the cells from all groups of mice were able to produce approx. 5 ng/ml of IL-10 on the 7^th^ day of the experiments (Fig 7B). However, the production slowly decreased along the experiment duration regardless of the nature of the treatment. At the same time, the concentration of IFN-γ in these supernatants did not exceed 1 ng/ml, even in the group of mice treated only with LOS. This production is likely to result from an indirect activation of T cells by LOS-stimulated myeloid cells associated with innate mediators of immunity.

### PmB-mediated modulation of LOS-inhibition of lung colonization by *i*.*v*. inoculated LLC cells

**Fig 8:**
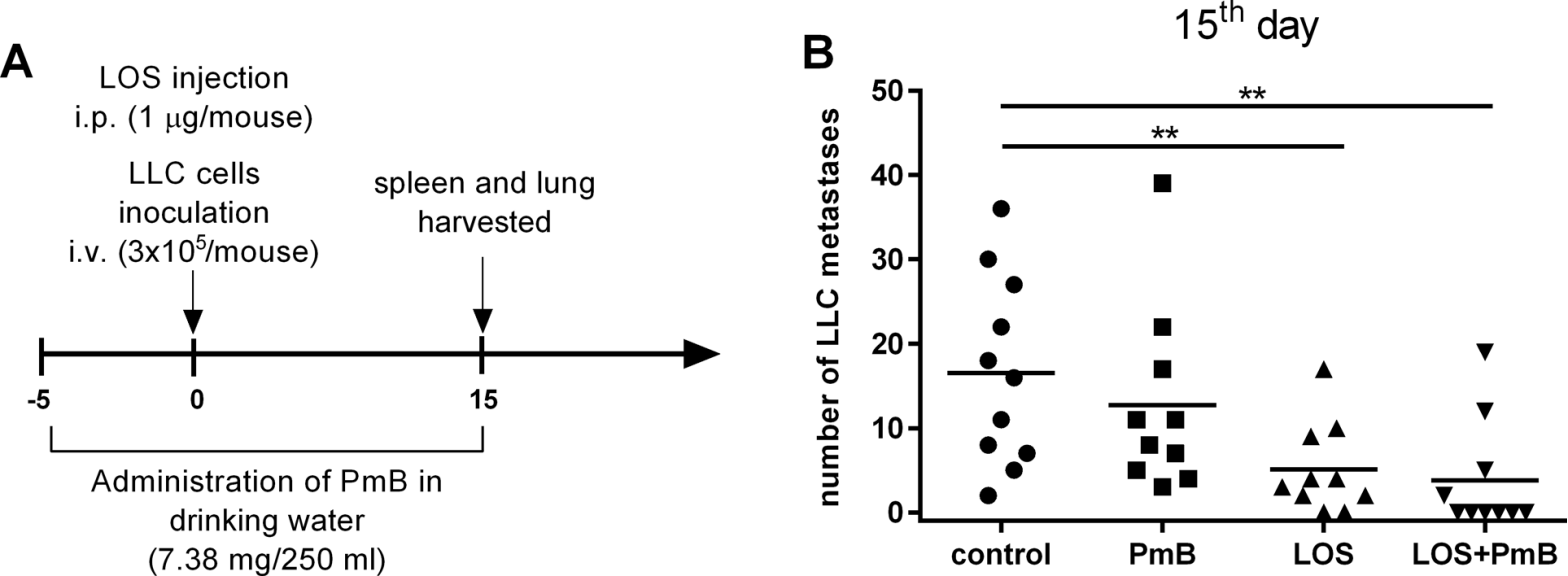
A. Mice treatment schedule. An administration of polymyxin B (7.38 mg/250 ml in drinking water) was started on the 5^th^ day before the i.v. injection of 3x10^5^ LLC cells and continued throughout the experiment duration. Mice were injected with LOS (1μg/mouse) i.p. one hour before cell injection. On the 15^th^ day after injection, material were harvested and number of lung metastases was determined. B. Number of the LLC metastasis in the lung obtained from treated mice on the 15^th^ day of experiment. To calculate the mean, 10–11 mice of each group of two experiments were tested. The relationship between groups was calculated for n>11 using Mann-Whitney *U* test. differences with a p<0.01 were regarded as significant (**p<0.01—calculated vs untreated mice).

The objective of this study was to analyze the effect of PmB on biological activity of LOS *E*.*coli* B *in vitro* and *in vivo* using B16-melanoma model. Despite the fact that the observed biological effect of the PmB + LOS combination has reduced the number of metastases in melanoma model we did not observed significant changes in the systemic specific response. Therefore we decided to use PmB for modulation of the anti-metastatic effect of LOS in the second model with inoculation of LLC cells. For this purpose, PmB was administered in drinking water starting 5 days prior to the intravenous inoculation of LLC cells. Likewise in B16 model, the main task of the treatment was to reach the highest environmental conditioning before LOS *i*.*p*. application (Fig 8A). The macroscopic metastases number in particular groups was analyzed on the 15th day of the experiments. The administration of PmB alone, resulted in similar number compared to control. Whereas, the application of LOS caused a statistically significant (p<0.01) decrease in the mean number of metastases. Statistically significant decrease in metastases number was also observed in the group of mice preconditioned by PmB and subsequently treated with LOS (LOS + PmB) (Fig 8B). Although the experiments involving B16- and LLC-models showed differences in case of statistically significant response on LOS or PmB applied separately, the use of both compounds together resulted in similar diminish of metastatic numbers when they were estimated on 14 or 15 days of experiments.

### Effect of PmB on phenotype alterations and induction of spleen cells from LLC mice model reactivity by LOS administration

**Fig 9:**
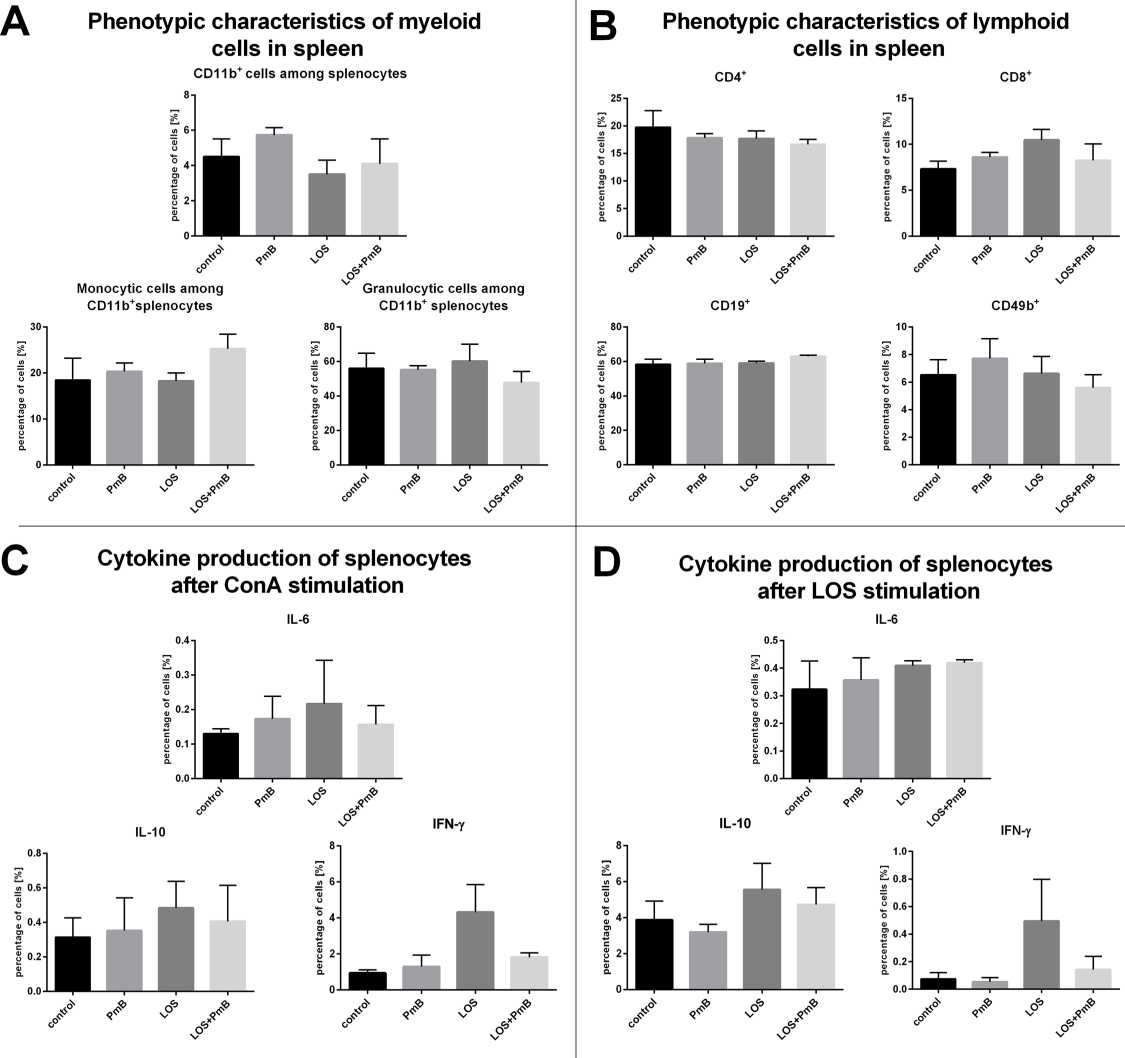
Phenotypic characteristics of myeloid (A) and lymphoid cells (B) in spleen. Spleen cells from mice treated with LOS and/or PmB were incubated with CD11b-PerCP-Cy5.5, B220-APC, Ly6G-APC-Cy7, Ly6C-PE, MHCII-FITC or anti-CD4-APC, anti- CD8-PE-Cy7, anti-CD49b-PE and anti-CD19-FITC. Phenotype analysis was carried out using the Becton Dickinson FACSCalibur apparatus with Cell Quest Software. To calculate the mean, three mice of each group were tested. The percentages of myeloid cells in healthy mice were: CD11b^+^—3.50%, monocytic cells among CD11b^+^ cells—30.50%, granulocytic cells among CD11b^+^ cells—30.90%. The percentages of lymphoid cells in healthy mice were: CD4^+^—17.43%, CD8^+^ 9.26%- CD19^+^—61.67%, CD49b^+^—8.00%. Cytokine production by spleen cells after ConA (C) or LOS (D) stimulation. The concentration of IL-6, IFN-γ and IL-10 was measured in supernatants collected from above splenocytes (2x10^6^ cells/ml) incubated for 48h with ConA (0.5 μg/ml) or LOS (1 μg/ml), using commercially available ELISA kits (BD Bioscience or eBioscience). To calculate the mean, three mice of each group were analyzed. Cells from healthy mice stimulated with ConA produced 1.07 ng/ml of IFN-γ, 0.10 ng/ml IL-6 and 0,23 ng/ml of IL-10. Cells from healthy mice stimulated with LOS produced 0.01 ng/ml of IFN-γ, 0.33 ng/ml IL-6 and 3.52 ng/ml IL-10.

During the next step of our research on PmB modulation of the LOS activity in LLC mice model, the phenotype alteration and induction of spleen cells reactivity were analyzed. The small percentage (4–6%) of CD11b^+^ cells subpopulation were found in spleens (Fig 9A). This population was further divided into Ly6C^hi^Ly6G^-^ (monocyte-derived splenocytes) and Ly6C^lo^ Ly6G^hi^ cells (granulocyte-derived splenocytes). The first subpopulation represented approx. 18% of CD11b^+^ cells in mice treated with LOS, and its size slightly increased to 25% after treatment with LOS + PmB. The second subpopulation, increased after LOS administration and reached 60% of CD11b^+^ cells. While, in mice conditioned with PmB + LOS the percentage of Ly6C^lo^ Ly6G^hi^ cells diminished to 50%.

In the experiments no significant changes in the percentage of CD19^+^, CD4^+^, CD8^+^, CD49b^+^ splenic subpopulations (Fig 9B) were found. A slight increase in CD8^+^ cell percentage after administration of LOS and in the percentage of CD49b^+^ cells after administration of PmB was observed compared to the control. While, slight decrease of both subpopulation was observed in mice treated with LOS + PmB.

Despite minimal alterations in the spleen cell phenotype we have estimated the level of cell reactivity. For this purpose, splenocytes were stimulated *ex vivo* with ConA (Fig 9C) or LOS (Fig 9D), and the concentrations of IL-6, IFN-γ, and IL-10 in the supernatants from the aforementioned cultures were measured. Likewise in B16 model, the small level of IL-6 production proved to be very similar regardless of the type of *in vivo* treatments. The highest production of IFN-γ was found in group treated with LOS in both types of *ex vivo* stimulations. However, the level of production was different (4 μg/ml after ConA stimulation, and 0,4 μg/ml after LOS stimulation). Besides only in this group of mice the statistically significant (*p*<0.05) changes in cytokine concentration were noticed. By contrast, the IL-10 concentration amounted to approx. or 0,5 ng/ml after ConA or 8 ng/ml after LOS stimulation and somewhat varied due to nature of treatment.

## Discussion

The objective of this study was to analyze the effect of polymyxin B (PmB) on biological activity of rough analog of LPS referred to as lipooligosaccharide (LOS) *E*.*coli* B *in vitro* and *in vivo*.

In *in vitro* studies, we observed the neutralizing effect of PmB on the biological activity of LOS. In view of fact that the monocyte-macrophage J774A.1 cell line has been often utilized for *in vitro* estimation of pro- and anti-inflammatory activity of various compounds including LPS [17, 18], we used this cell line to analyze the interaction between PmB and LOS that consists of the core oligosaccharide and lipid A [7]. Likewise in other immunological studies, in our experiments, PmB used in high concentrations (10–50 μg/ml) did not affect the cell proliferation of both tumor and endothelial cell lines by more than 5%. Addition of PmB to the culture of J774A.1 cells stimulated with LOS, resulted in diminished production of inflammatory cytokines (IL-6, IL-10, TNF-α) but only when its concentration in the culture exceeded that of LOS by several times. A similar effect of PmB was presented by Wolfert’s group [10], in which the addition of PmB (30 μg/ml) to the cell culture prior to incubation with *E*. *coli* O55:B5 LPS (10 ng/ml) resulted in considerable decrease in TNF-α production [10].

In order to confirm whether presence of PmB in cell cultures affected LOS stimulation of other myeloid cells, we employed bone marrow-derived dendritic cells (BM-DCs)—the cells generally thought to be programmed for strong response to stimulation with LPS. In consequence of such stimulation we have observed the up-regulation of cell-surface antigen expression and secretion of proinflammatory cytokines eg. TNF-α, IL-12, or IL-6. The treatment with PmB resulted in diminished production of inflammatory cytokines by these cells and the expression of CD40, CD80, CD86 and MHC class II molecules on surfece of BM-DC. The addition of PmB to LOS-stimulated cells, at the compound ratio of 1:1 prevented on increase of the cell surface antigen expression, but only at compound ratio of 10:1, it additionally reduced cytokine production to the control level.

Evaluation of PmB-mediated modulation of LOS-inhibition *in vivo* appeared to be much more complex. Polymyxin B is a natural potent antibiotic that is able to bind and neutralize LPS, preventing its effects in animal models [19]. However, there is evidence indicating that administration of polymyxins is associated with considerable nephrotoxicity and neurotoxicity [20]. Like other compounds, PmB disclose the considerable cytotoxicity. In order to bypass its toxic activity, in our experimental metastases model PmB was administered orally in drinking water during the experiment, despite very low absorption of PmB from intestinal mucosa. Nevertheless, one of the recent papers showed that intranasal co-administration of polymyxins with protein antigens enhances specific humoral immunity in mucosal and systemic response, including immunological memory in mouse models [21]. The researchers also observed that the injection of LPS modulated tumor progression in a dose-dependent manner. A low dose of LPS increased lung tumor burden, whereas a high dose of LPS decreased tumor progression in lungs [21].

In respect to our, *in vitro* data illustrating that PmB mediated the neutralization of LOS effect, we analyzed the use of PmB for modulation of the anti-metastatic effect of LOS in B16 and LLC models. The results suggest that exposure of mice to permanent PmB administration had an indirect influence on immune cell response towards LOS. In both *in vivo* models, a statistically significant decrease in number of metastases in mice treated with PmB and LOS (PmB + LOS) was observed. Thus, we hypothesize that the advanced reduction of the number of metastases observed in the group treated with PmB + LOS combination was elicited by changes in the immune activity rather than by creation of the *ad hock* PmB-LPS complexes. Nevertheless, some paper described molecules (eg. hsp) which can protect small amounts of LPS from binding to PmB and ensure its delivery to cellular receptors [22].

In order to analyze the relationship between PmB-preconditioning and i.p. LOS administration we harvested the peritoneal cavity (PC) cells from tumor-bearing mice. Murine PC contains specialized immune cells, mainly macrophages (Mφs), which are characterized by expression of surface F4/80 glycoprotein and CD11b integrin. Ghosn’s group, based on cell size differences, divided PCMφs into large and small Mφs. The population determined as the large Mφs represented 90% of the PCMφs expressing high levels of CD11b and F4/80 in unstimulated animals, but did not express MHC class II antigens. On the other hand, small Mφs expressed lower levels of CD11b and F4/80, but high level of MHC class II. The population of large Mφs disappeared rapidly from the PC as the aftermath of LPS stimulation. However, the small Mφs, which were predominant in the peritoneal cavity after LPS stimulation did not derive from large Mφs, but rather from blood monocytes that rapidly entered the PC after the stimulation and subsequently differentiated into mature small peritoneal Mφs within 2 to 4 days [23]. In most mouse models, the time interval, in which the immune cells retain increased activity after treatment with LPS does not exceed 3 to 4 days [24]. In contrast to them, we harvested peritoneal cells from B16 bearing mice on the 7th, 14th, and 21st day of experiments. The most significant differences between the controls and groups of mice treated with PmB or treated with LOS +/- PmB were observed on the 7th day of the experiments even before the appearance of metastases. At the same time the detectable production of IL-1β and TNF-α (but only in mice which obtained LOS+PmB) was noticed. In the experiments reported by other investigators high serum concentration of the proinflammatory cytokines was found several hours after application of LPS [24]. Therefore, our observations of prolonged presence of small concentrations of IL-1β and TNF-α in blood, even 7 days after the usage of LOS (and especially the detection of IL-6 on the 21^st^ day), could be the results of a secondary action of myeloid cells in antitumor response than a direct effect of LOS administration.

Innate immune response leads to activation of acquired immunity. However, this response depends on several factors, such as the nature and power of the stimuli, as well as the number of their applications and length of their presence in the organism. There is evidence suggesting that the activity of macrophages and DCs, which respond to LPS stimulation can be changed by signals derived from T cells and/or other cells [25,26]. Thus, it seemed to be very interesting to analyze the level of splenocytes activity in the treated mice. For this purpose, the phenotypic characteristics of spleen cells and production of cytokines (IL-6, IL-10, IFN-γ) by these cells after stimulation with ConA or LOS were investigated in both metastases models. When splenocytes obtained from treated mice were stimulated *ex vivo* with LOS, the cells from all groups of mice were able to produce high amount of IL-10. However, the production of IL-10 was time-dependent and slowly decreased along the experiment duration regardless of the nature of the treatment (B16 model) and treatment-dependent (LLC model). At the same time, the concentration of IFN-γ in analyzed supernatants did not exceed 1 ng/ml, even in the group of mice treated only with LOS. This production presumably result from an indirect activation of T cells by LOS-stimulated myeloid cells associated with innate mediators of immunity—macrophages and NK cells, and auxiliary cells such as stromal and endothelial cells [27]. However, there is evidence which shows that mice infected with pathogens or challenged with TLR ligands have a small subpopulation of IFN-γ-producing CD19^+^ spleen cells activated during the early stage of the immune response [28]. Although LOS administration to metastase-bearing mice is able to induce lymphocyte activity, this response did not prove to be responsible for the anti-metastatic effects. As reported by other investigators a bystander stimulation of T cells observed after injection of LPS (TLR4 agonists) caused a phenotypic activation of these cells [27]. In our experiments we did not observe such a result after treatment with LOS, or in the case of PmB-conditioned mice.

## Conclusions

The aim of this study was to analyze the remote influence of polymyxin B on ability of the *E*. *coli* B lipooligosaccharide to inhibit lung in experimental metastasis. Taking together, although prolonged application of PmB *in vivo* was not able to elicit strong reactivity of the immune cells, its presence in environment of the LOS-treated mice modulated the trigger of the immune response. Therefore, we postulate, that peritoneal–and/or blood–derived myeloid cells, which responded to LOS administration with the release of the cytokines mobilizing antitumor cells immunity played crucial role in the process.
